# iTRAQ Proteomics Identified the Potential Biomarkers of Coronary Artery Lesion in Kawasaki Disease and In Vitro Studies Demonstrated That S100A4 Treatment Made HCAECs More Susceptible to Neutrophil Infiltration

**DOI:** 10.3390/ijms232112770

**Published:** 2022-10-23

**Authors:** Ken-Pen Weng, Kuang-Jen Chien, Shih-Hui Huang, Lien-Hung Huang, Pei-Hsien Lin, Yuyu Lin, Wei-Hsiang Chang, Chun-Yu Chen, Sung-Chou Li

**Affiliations:** 1 Department of Pediatrics, Kaohsiung Veterans General Hospital, Kaohsiung 813414, Taiwan; 2 School of Medicine, National Yang Ming Chiao Tung University, Taipei 11221, Taiwan; 3 Department of Nursing, Fooyin University, Kaohsiung 83102, Taiwan; 4 Department of Neurosurgery, Kaohsiung Chang Gung Memorial Hospital and Chang Gung University College of Medicine, Kaohsiung 83301, Taiwan; 5 Genomics and Proteomics Core Laboratory, Department of Medical Research, Kaohsiung Chang Gung Memorial Hospital and Chang Gung University College of Medicine, Kaohsiung 83301, Taiwan; 6 Department of Food Safety/Hygiene and Risk Management, Medical College, National Cheng Kung University, Tainan 701401, Taiwan; 7 Department of Pediatrics, Chi Mei Medical Center, Tainan 71004, Taiwan; 8 Center for Mitochondrial Research, Kaohsiung Chang Gung Memorial Hospital and Chang Gung University College of Medicine, Kaohsiung 83301, Taiwan

**Keywords:** Kawasaki disease, coronary artery lesion, biomarker, proteomics, S100A4, endothelial migration, permeability, infiltration

## Abstract

Coronary artery lesions (CAL) are a major complication of Kawasaki disease (KD). The early prediction of CAL enables the medical personnel to apply adequate medical intervention. We collected the serum samples from the KD patients with CAL (*n* = 32) and those without CAL (*n* = 31), followed by a global screening with isobaric tagging for relative and absolute quantification (iTRAQ) technology and specific validation with an enzyme-linked immunosorbent assay (ELISA). iTRAQ identified 846 proteins in total in the serum samples, and four candidate proteins related to CAL were selected for ELISA validation as follows: Protein S100-A4 (S100A4), Catalase (CAT), Folate receptor gamma (FOLR3), and Galectin 10 (CLC). ELISA validation showed that the S100A4 level was significantly higher in KD patients with CAL than in those without CAL (225.2 ± 209.5 vs. 143.3 ± 83 pg/mL, *p* < 0.05). In addition, KD patients with CAL had a significantly lower CAT level than those without CAL (1.6 ± 1.5 vs. 2.7 ± 2.3 ng/mL, *p* < 0.05). Next, we found that S100A4 treatment on human coronary artery endothelial cells (HCAECs) reduced the abundance of cell junction proteins, which promoted the migration of HCAECs. Further assays also demonstrated that S100A4 treatment enhanced the permeability of the endothelial layer. These results concluded that S100A4 treatment resulted in an incompact endothelial layer and made HCAECs more susceptible to in vitro neutrophil infiltration. In addition, both upregulated S100A4 and downregulated CAT increased the risk of CAL in KD. Further in vitro study implied that S100A4 could be a potential therapeutic target for CAL in KD.

## 1. Introduction

Kawasaki disease (KD) is liable to be complicated by the coronary artery lesion (CAL) and is the primary etiology of acquired heart disease in the pediatric population [1]. The incidence of CAL is about 15–25% in KD patients without adequate treatment [2] and 4% in those with intravenous immunoglobulin (IVIG) therapy [1,3]. Other rare complications of KD may include medium-sized aneurysms, peripheral gangrene, peripheral facial nerve palsy, sensorineural hearing loss, etc. [1].

CAL during the acute stage of KD can range from mild dilation to severe aneurysms. A large quantity of the literature surrounding this has shown potential predictors of CAL development in KD, including demographic, clinical, and laboratory variables. The risk factors of CAL in KD patients are as follows: IVIG resistance, anemia, hypoalbuminemia, leukocytosis with predominant neutrophil count, high C-reactive protein (CRP) levels, male sex, and age younger than 1 year or older than 6 years [4,5,6,7,8,9]. Weng et al. showed that the risk factors of acute CAL also included neutrophil count, the second dose of IVIG treatment, and platelet count, while those of chronic CAL included age, the first dose of IVIG treatment, and band count [10]. The study by Popper et al. suggested that the neutrophil activation state and apoptosis might contribute to the pathogenesis of KD [11]. An influx of neutrophil development in the early stage (7–9 days after KD onset), with a rapid transition into large mononuclear cells, can exit in concert with lymphocytes and immunoglobulin A plasma cells [12,13]. Beiser and his colleagues constructed a sequential risk classification instrument based on easily measured baseline laboratory data and body temperature, including neutrophil count, and their results demonstrated that no low-risk patient based on this instrument developed CAL [5]. Masuda et al. reported the following risk factors associated with giant CAL in KD: male sex, recurrent KD, an early or delayed IVIG administration, the detection of CAL at initial echocardiography, and IVIG resistance [14]. These previously reported studies are primarily based on clinical results [4,5,6,7,8,9,10,14], and further in vitro or in vivo studies are required to elucidate these risk factors of CAL in KD.

Isobaric tagging for the relative and absolute quantification (iTRAQ) of gel-free proteomics has emerged as a powerful tool for discovering candidate protein biomarkers and has shown feasibility in identifying disease markers of KD [15]. In this study, we first enrolled KD patients with or without CAL. Then, we applied iTRAQ to globally screen serum samples of KD patients, detecting as many serum proteins as possible. The following ELISA measurement validated the candidate biomarkers of CAL in KD. With an in vitro cell model applied in a previous study [16], we also investigated the roles of S100A4 in the pathogenesis of CAL related to KD.

## 2. Results

### 2.1. The Overview of Participants

As shown in Table 1, 32 KD patients with CAL (CAL+KD) and 31 KD patients without CAL (CAL-KD) were enrolled in this study. There was no significant difference between CAL+KD and CA-KD sets in terms of age, sex, height, and weight factors. The subjects in the CAL+KD set had a significantly higher inpatient day, Z score of the left coronary artery (LCA) (Z-LCA), and Z score of the right coronary artery (RCA) (Z-RCA) than those in the CAL-KD set.

### 2.2. Global Screening of Serum Proteins with iTRAQ Assay

**Figure 1:**
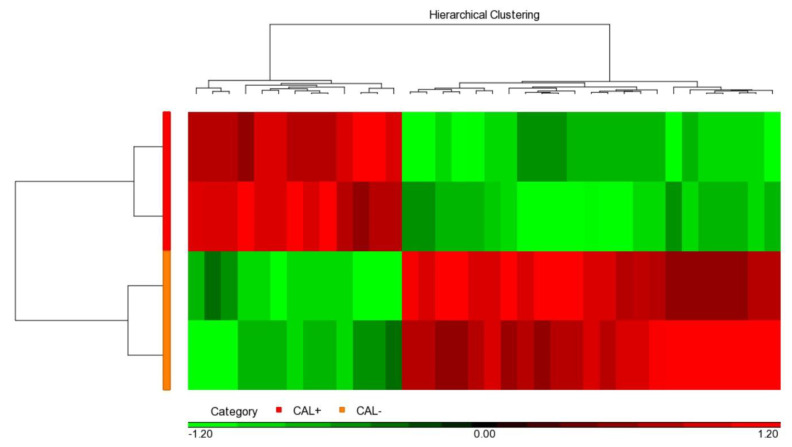
Heat map illustrated 36 significant serum proteins. Among the 846 proteins globally detected in two CAL+KD and two CAL−KD serum samples, 36 were significantly (*p*-value < 0.05) and differentially abundant. The red and green pixels denote up-regulation and down-regulation in CAL+KD samples, respectively. CAL—coronary artery lesion; KD—Kawasaki disease.

We used the iTRAQ assay to determine and compare protein profiles between the two pooled CAL+KD and two pooled CAL-KD serum samples. As a result, 846 proteins were identified and based on the two parameters: protein and peptide identifications FDR ≤ 0.01 and the protein identified with at least one unique peptide. When the “unique peptide” criterion was set as two, the number of the identified protein was 638. The detailed and valuable information on detected proteins is accessible in Supplementary Table S1. The protein abundance profile was further analyzed with Partek. It turned out that there were 36 proteins differentially abundant between the CAL+KD and CA-KD samples (*p*-value < 0.05, Figure 1, Supplementary Table S2). In addition, 13 and 23 were up-regulated and down-regulated in the CAL+KD samples, respectively.

### 2.3. Specific Validation of Serum Proteins with Enzyme-Linked Immunosorbent Assay (ELISA)

**Figure 2:**
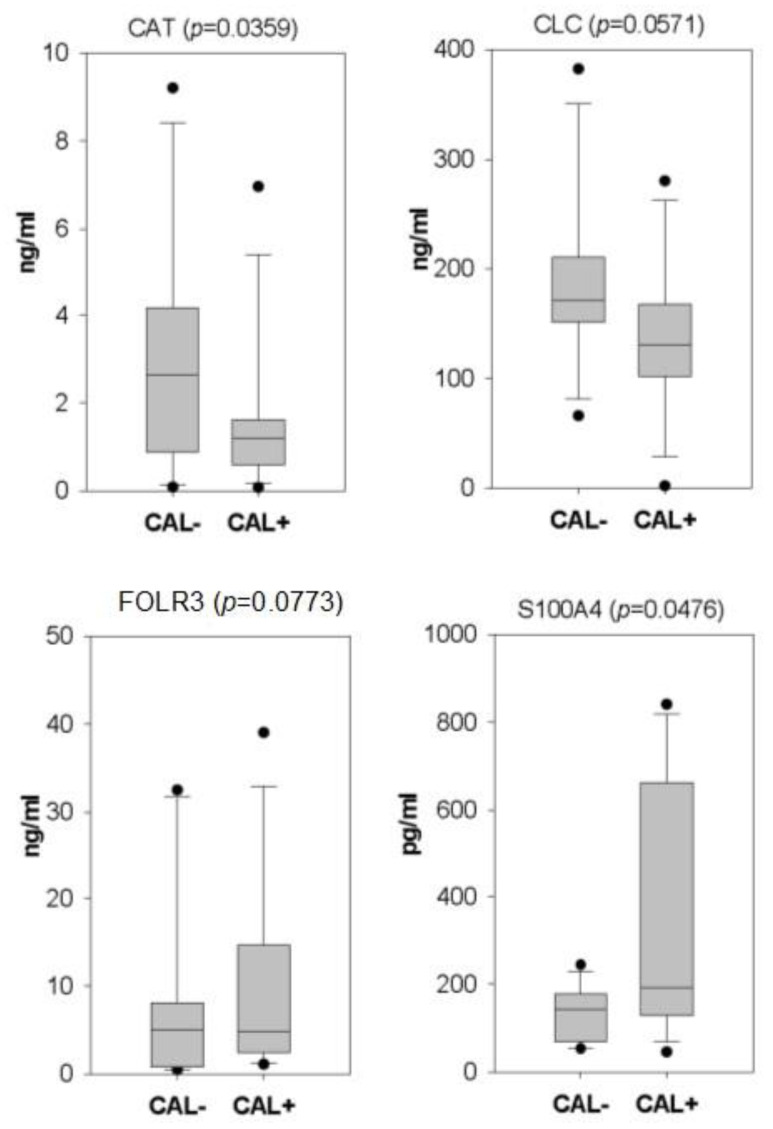
Box plots illustrate the ELISA validation results of four candidate CAL biomarker proteins. Following iTRAQ assay, four candidate serum proteins were validated with ELISA on all serum samples. With statistical significance, CAT and S100A4 kept higher levels in CAL-KD and CAL+KD samples, respectively. CAT—catalase; CLC—galectin 10; FOLR3—Folate receptor gamma; S100A4—S100 calcium-binding protein A4; CAL—coronary artery lesion; KD—Kawasaki disease.

Among the detected serum proteins with a variation >1.5-fold, we used the literature search to select the ones which could be associated with CAL in KD. As a result, four serum proteins, including catalase (CAT), galectin 10 (CLC), folate receptor gamma (FOLR3), and S100 calcium-binding protein A4 (S100A4), were validated with ELISA in all the serum samples. As shown in Figure 2, CAT and S100A4 reached statistical significance. In addition, among the four examined proteins, S100A4 and FOLR3 kept higher levels in the CAL+KD samples. The remaining two proteins had higher levels in the CAL-KD samples.

### 2.4. S100A4 Reduced Cell Junction Protein

**Figure 3:**
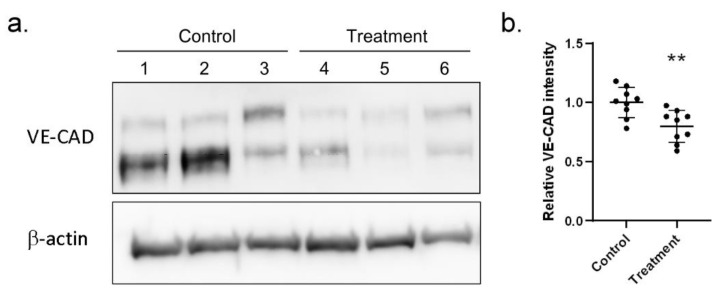
Western blot assay demonstrated reduced VE-CAD proteins with S100A4 treatment on HCAECs. We used a Western blot assay to examine whether S100A4 treatment (45 pg/mL) reduced the abundance of cell junction proteins (VE-CAD) on HCAECs. (**a**) One round of Western blot assay result (three replications). (**b**) By three independent rounds of assay (three rounds * three replications), a significant difference was reached. Data were presented as mean ± SD. ** denoted *p*-value < 0.01. VE-CAD—vascular endothelial cadherin protein; HCAECs—human coronary artery endothelial cells; S100A4—S100 calcium-binding protein A4.

Among the proteins validated with ELISA, we were especially interested in S100A4 since it could promote endothelial cell migration [17] and was first found to be a new biomarker related to KD. In the human coronary artery, endothelial cells form a compact layer, allow blood to flow and keep leukocytes from infiltrating into the tissue layer. Therefore, we wondered whether the elevated level of serum S100A4 weakened the endothelial cell junction, promoting endothelial cell migration, enhancing endothelial permeability and finally leading to leukocyte infiltration. To examine our hypothesis, we first examined the abundance of the vascular endothelial cadherin protein (VE-CAD, also called CD144) in human coronary artery endothelial cells (HCAECs) when treated both with and without the S100A4 protein. As shown in Figure 3a, S100A4 treatment lowered down the abundance of VE-CAD. In three independent assays, a significant (*p*-value < 0.01) reduction in VE-CAD was observed (Figure 3b). Therefore, S100A4 treatment repressed the abundance of VE-CAD, weakening the intensity of the endothelial cell junction.

### 2.5. S100A4 Promoted Endothelial Migration

**Figure 4:**
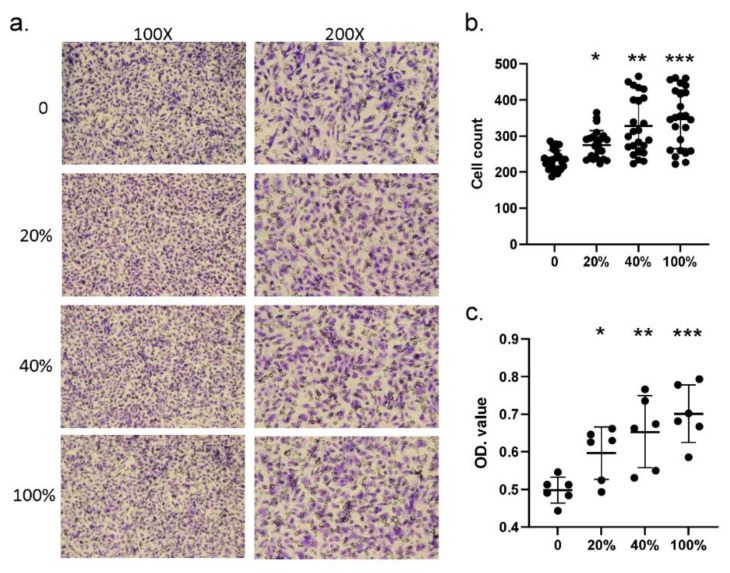
Trans-well assay showed that S100A4 promoted the migration of HCAECs. We used a trans-well kit to examine whether S100A4 affected the migration ability of HCAECs. (**a**) With the treatment of different S100A4 dosages, 45 pg/mL (20% of the average concentration on CAL+KD subjects), 90 pg/mL (40%), 180 pg/mL (80%), and 225 pg/mL (100%), HCAECs were subjected to a migration ability examination with a trans-well kit. (**b**) The HCAECs penetrating the insert membrane were quantified by staining and counting (three rounds * eight replications). (**c**) The cells were quantified by staining and measuring the OD values (three rounds * two replications). Data were presented as mean ± SD. *, ** and *** with a denoted *p*-value < 0.05, 0.01, and 0.001, respectively. S100A4—S100 calcium-binding protein A4; HCAECs—human coronary artery endothelial cells; CAL—coronary artery lesion; KD—Kawasaki disease; OD—optical density.

Next, we investigated whether S100A4 affected the migration ability by treating HCAECs with different dosages of the S100A4 protein. Figure 4a illustrates the result of one round of trans-well migration assays. With a higher concentration of S100A4 administrated, more HCAECs penetrated the insert membrane and migrated into the bottom side of the membrane. By three independent runs of assays, the S100A4 treatment significantly promoted the migration ability of HCAECs through the counting of migrating cells (Figure 4b). Moreover, such promotion effects demonstrated a dosage-dependent manner. In addition to counting the numbers of migrating cells, we also repeated the assays by collecting the cells at the bottom side, followed by measuring the optical density (OD) values. As shown in Figure 4c, consistent results were observed. Therefore, S100A4 treatment promoted the migration ability of HCAECs.

### 2.6. S100A4 Promoted the Permeability of the Endothelial Layer

**Figure 5:**
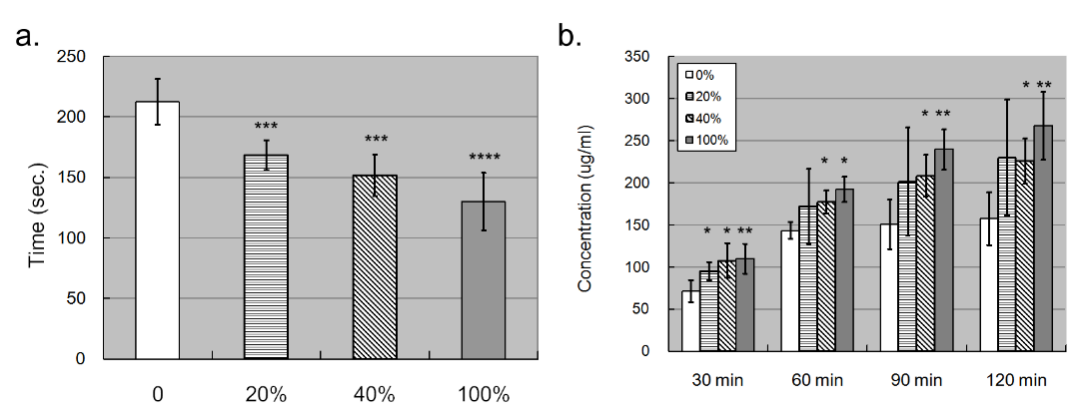
Trans-well assay showed that S100A4 promoted the permeability of the endothelial layer composed of HCAECs. We also used trans-well kits to examine whether S100A4 affected the permeability of the endothelial layer. (**a**) The permeability of the endothelial layer was quantified by counting how much time was needed for trypan blue to reach the lower chamber (*n* = 3). (**b**) The permeability of the endothelial layer was quantified by measuring the concentration of total proteins in the lower chamber (*n* = 3). Data were presented as mean ± SD. *, **, *** and **** with a denoted *p*-value < 0.05, 0.01, 0.001, and 0.0001, respectively. S100A4—S100 calcium-binding protein A4; HCAECs—human coronary artery endothelial cells.

Since the elevated S100A4 promoted endothelial migration, we wondered whether such migration lowered down the compactness of the endothelial layer, affecting its permeability. To examine our hypothesis, we conducted a permeability assay with the trans-well facility. We first put trypan blue dye in the upper chamber and observed how much time was needed for the trypan blue to reach the lower chamber. The less time needed denoted, the higher permeability. It turned out that the trypan blue needed significantly less time to penetrate the endothelial layer and reach the lower chamber with S100A4 treatment (Figure 5a). In addition, as observed in the migration assay, such an effect was also dosage dependent. To derive robust results, we repeated the permeability assay by replacing the trypan blue dye with FBS, which was added in the upper chamber. After a specific time, we measured the protein concentration in the lower chamber. The higher concentration that was detected denoted a higher permeability. Figure 5b demonstrates how significantly more FBS penetrated the endothelial layer with S100A4 treatment and reached the lower chamber in most assays. In addition, this effect demonstrated time-dependent and dosage-dependent manners. Therefore, S100A4 enhanced the permeability of the endothelial layer composed of HCAECs.

### 2.7. S100A4 Enhanced the Susceptibility of Endothelial Layer to In Vitro Neutrophil Infiltration

**Figure 6:**
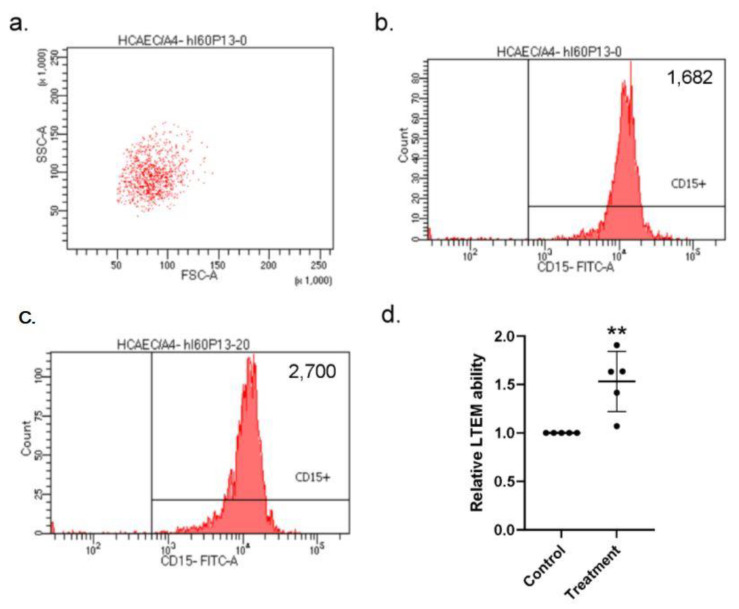
LTEM assay showed that the endothelial layer was more susceptible to in vitro neutrophil infiltration with S100A4 treatment. (**a**) To examine whether the weakened endothelial layer was more susceptible to neutrophil infiltration, we conducted the LTEM assay. (**b**,**c**) S100A4 treatment on the endothelial layer allowed more HL-60 cells to penetrate (2700 vs. 1682). (**d**) By three independent assays (three rounds * replications, one replication failed), more HL-60 cells penetrated the endothelial layer and a 1.5-fold higher LTEM ability was observed with S100A4 treatment. Each dot denoted the relative LTEM ability of one assay. Data were presented as mean ± SD. **with a denoted *p*-value < 0.01. LTEM—leukocyte trans-well endothelial migration; S100A4—S100 calcium-binding protein A4; HL-60—a neutrophil-like cell line.

Combining the current results, we hypothesized that the elevated S100A4 could enhance the susceptibility of the endothelial layer to in vitro neutrophil infiltration, which is similar to the in vivo behavior of CAL in KD patients. To examine our hypothesis, we conducted in vitro leukocyte trans-well endothelial migration (LTEM) assays by referring to the previous studies [15,18,19] with the major modification that it was HCAECs, rather than leukocytes that were treated with the S100A4 protein. We first gated HL-60 (a neutrophil-like cell line) cells with specific SCC-A and FSC-A values (Figure 6a) and with a flow cytometry. After one round of the LTEM assay, more HL-60 cells (2700 vs. 1682) penetrated the endothelial layer with S100A4 treatment (Figure 6b,c). By three independent assays, S100A4 treatment on the HCAECs conferred HL-60 cells with a 1.5-fold in vitro LTEM ability (Figure 6d). Therefore, S100A4 enhanced the susceptibility of the endothelial layer to in vitro neutrophil infiltration, which was consistent with the previous findings in this study.

## 3. Discussion

Among the serum proteins identified with iTRAQ technology, four candidate proteins were selected as follows: CLC, FOLR3, CAT, and S100A4. CLC, also named galectin-10, was selected since it was reported to play an important role in the anergy and suppressive function of CD25^+^ Treg cells [20]. Additionally, Treg cells were required for the development of KD vasculitis [21]. During the formation of CAL in KD, the attachment between the leukocyte and vessel endothelial cells was conducted through cell adhesion. Moreover, FOLR3 had the potential to participate in the cell adhesion [22]. In addition, FOLR3 was often observed in the patients with other inflammatory disease [23]. However, only upregulated S100A4 and downregulated CAT significantly increased the risk of CAL in KD patients after ELISA validation. To the best of our knowledge, this is the first study using iTRAQ to identify the potential biomarkers of CAL in KD.

CAT has the potential to protect cells from the damage caused by hydrogen peroxide [24]. Oxidative stress by reactive oxygen species (ROS) has been reported to contribute to the formation of CAL in KD [25,26,27,28]. Cheung et al. reported that KD patients with CAL had the carotid intima-media thickening and stiffening associated with oxidative stress [25]. Ishikawa and his colleagues found that the fever duration of KD was positively associated with the risk of oxidative stress-induced endothelial dysfunction and suggested that oxidative stress was strongly associated with endothelial dysfunction in young children with KD [28]. Moreover, Niwa et al. concluded that the intermediate production of oxygen in neutrophils from KD patients were reduced to the control level in the presence of superoxide dismutase and catalase [29]. Moreover, Uchida suggested that increased oxidative stress in KD evoked a reactive increase in antioxidant enzymes, including CAT [30]. The beneficial managements against oxidative stress in KD were also reported previously [31,32,33,34]. These previous studies implicated that CAT could play a protective role against CAL formation in KD, which was consistent with our observation that down-regulated CAT acts as a risk factor of CAL. Therefore, the important role of CAT as an antioxidant against oxidative stress in the pathogenesis of KD deserves further investigations.

S100A4 was selected for these further investigations since it was reported to promote the migration ability of endothelial cells, thereby enhancing tumor metastasis [17]. Additionally, the role of S100A4 in the pathogenesis of KD has not been investigated previously. S100A4 is a potent trigger of inflammatory molecules, such as interleukin (IL)-1, IL-6, tumor necrosis factor (TNF)-a, and matrix metalloproteinases, which are involved in autoimmune disease developments, such as allergies, rheumatoid arthritis, systemic sclerosis, and psoriasis, etc. [35]. The effect of S100A4 on the coronary artery has been demonstrated in both in vivo and in vitro animal studies [36]. Brisset et al. reported that S100A4 was highly expressed by smooth muscle cells (SMCs) of intimal thickening but absent in the normal porcine coronary artery media [36]. Therefore, S100A4 is a marker of porcine intimal thickening development in the in vitro study [36]. In addition, human S100A4 was also markedly expressed in SMCs of coronary artery lesions but barely detectable in the coronary artery media [36]. Sakic and his colleagues used a mouse model of atherosclerosis to demonstrate the neutralization of extracellular S100A4 and decreased areas of the atherosclerotic lesions [37]. Both the two previous animal studies partially support our findings about the up-regulated role of S100A4 in CAL complicated with KD.

Extra-cellular cytokines usually perform their regulation potential on target cells through the membrane receptors of the target cells. For example, serum S100A8 and S100A9 heterodimer bind to the Toll-like receptor 4 on the neutrophil membrane to trigger the following inflammatory reactions [38]. Although previous studies concluded that S100A4 could interact with the following membrane receptors, including RAGE, EGFR, ERBB2, TLR4, and IL10R [39,40], these findings were derived from the nervous system or cancer-related studies. The membrane receptor of S100A4 on vascular endothelial cells is not yet clear. Therefore, the issue of whether the effects of S100A4 are dependent on its receptor in endothelial cells merits further investigation.

Our in vitro study of S100A4 provided evidence of endothelial damage in the intima with the subsequent formation of CAL. Therefore, S100A4 might be a new therapeutic target in KD patients, especially IVIG-resistant KD patients. Further studies are required to clarify the interaction of S100A4 with other cytokines to understand the pathogenesis of CAL in KD.

There are some limitations to this study. This is a single-center investigation of a small number of KD patients. The small sample size in the measurement of CAT and S100A4 levels may affect the statistical power for the detection of significant differences between KD patients with/without CAL. Therefore, a multicenter study with large sample size is suggested.

## 4. Materials and Methods

### 4.1. Participant Enrollment and Serum Collection

We enrolled KD patients between 2016 and 2021 at the Department of Pediatrics, Kaohsiung Veterans General Hospital, Taiwan. KD patients met diagnostic criteria, including fever for at least 5 days, nonpurulent conjunctivitis, extremity edema, oral mucosal changes with red lips and strawberry tongue, cervical lymphadenopathy, a polymorphous skin rash and Bacillus Calmette-Guérin (BCG) site erythema. [41] In addition, they also underwent a two-dimensional echocardiography to calculate their Z-LCA and Z-RCA scores [42]. In summary, the diameters of LCA and RCA were measured between the inner echo edges during the early diastolic phase at the end of the T wave. LCA was measured midway between the orifice and the bifurcation of the circumflex coronary artery. RCA was measured from 3 to 5 mm distal to the orifice. The Z-score value of LCA and RCA was then calculated from the following equation: Z-score = [ln (coronary artery) − β1 − β2 × ln (body surface area)]/standard error. CAL was based on a Z score in the acute stage and was defined as a Z-LCA or Z-RCA score ≥ 2 or within 2 weeks of illness.

In total, 32 KD subjects with CAL (CAL+KD, either Z-LCA ≥ 2 or Z-RCA ≥ 2) and 31 KD subjects without CAL (CAL-KD, both Z-LCA < 2 and Z-RCA < 2) participated in this study. The exclusion criteria of KD patients were as follows: (1) no serum samples before IVIG treatment in the acute stage, (2) missing laboratory data, and (3) no IVIG treatment or initial IVIG treatment beyond 10 days of the fever. The blood samples were applied for serum collection immediately, and the collected serum samples were stored at −80 °C in a refrigerator before the assays. This study was approved by the institutional ethics board (IRB approval number VGHKS19-CT2-22) of the Kaohsiung Veterans General Hospital. All subjects or their guardians signed the informed consent form, and all protocols were carried out in accordance with the relevant guidelines and regulations.

### 4.2. iTRAQ Gel-Free Proteomics

The protein profiles were globally determined with iTRAQ gel-free proteomics and specifically validated with ELISA. We conducted the iTRAQ gel-free proteomics assay by referring to the previous study [18]. In summary, 24 serum samples from KD patients (12 with CAL and 12 without CAL) were first subjected to a high-abundant protein depletion with the Pierce Top 12 Abundant Protein Depletion Spin Columns (85165, Thermo, Waltham, MA, USA) to remove highly abundant proteins, including albumin, immuno-globulin (Ig)G, IgA, IgM, α1-acid glycoprotein, α1-antitrypsin, α2-macroglobulin, apolipoprotein A-I, apolipoprotein A-II, fibrinogen, haptoglobin, and transferrin. Next, the six serum samples from the KD patients with CAL were evenly pooled to generate one pooled serum library. Meanwhile, the six serum samples from KD patients without CAL were also evenly pooled to generate one pooled serum library. As a result, two pooled serum samples with CAL and two pooled serum samples without CAL were collected.

The four pooled serum libraries were subjected to peptide labeling with the iTRAQ Reagents Multiplex Kit (4352135, Sciex, Framingham, MA, USA) and analysis with a LC/Q-Exactive Orbitrap MS (Thermo, Waltham, MA, USA) for 24 h. By referring to the MASCOT 2.5 database (Matrix science), the relative abundances of detected proteins were determined.

### 4.3. ELISA Validation for Selected Candidate Proteins

Among the proteins detected by iTRAQ gel-free proteomics, we selected four candidate proteins for ELISA validation with the following kits: CAT (CSB-EL020632HU, Cusabio, Houston, TX, USA), S100A4 (CY-8086, MBL, Woburn, MA, USA), FOLR3 (ab267613, Abcam, Cambrodge, UK), and CLC (NBP2-75319, Novus, Centennial, CO, USA). The procedure for ELISA was performed according to the manufacturer’s instructions.

### 4.4. Cell Culture and Treatment

In this study, two kinds of cell lines were cultured and applied. The first one was the primary human coronary artery endothelial cell (HUVEC, CC-2585, Clonetics, Lonza Walkersville, Walkersville, MD, USA), and the second one was the neutrophil-like cell (HL-60, No. 60027, BCRC, Hsinchu, Taiwan). They were cultured and maintained by referring to the previous study [19]. HL-60 cells were treated with DMSO for 24 h to induce neutrophil morphology before the assays. In this study, the average concentration of the serum S100A4 protein in the CAL samples was 225.16 pg/mL. Therefore, we had HUVECs treated with human S100A4 recombinant proteins (4137-S4, R&D systems, Minneapolis, MN, USA) by the following different dosages: 45 pg/mL (20% of the average concentration in CAL+KD samples), 90 pg/mL (40%), 180 pg/mL (80%), and 225 pg/mL (100%) for 24 h, followed by the assays.

### 4.5. Cell Lysate Preparation and Western Blotting Assay

HCAEC cell lysates were homogenized in the protein extraction solution (17081, iNtRON Biotechnology, Seongnam, Korea), and the protein samples were quantified using the Bradford protein assay (5000002, Bio-Rad, Hercules, CA, USA). A total of 20μg from the protein sample was mixed with the sample buffer (161-0747, Bio-Rad, Hercules, CA, USA) and boiled for 10 min. Protein samples were subjected to 10% SDS-polyacrylamide gel electrophoresis and a Western blot analysis using primary antibodies against VE-cadherin (MA1-198, Thermo Fisher, Waltham, MA, USA) and β-actin (8226, Abcam, Cambrodge, UK). Bound IgG antibodies were detected with horseradish peroxidase-labeled secondary antibodies (31430, Thermo Fisher, Waltham, MA, USA). Thereafter the chemiluminescence was used to detect the signals (GERPN2235, GE, Boston, MA, USA), and images were acquired by a GE Image Quant LAS 4000 and Image Quant LAS 4000 Control Software.

### 4.6. Migration Assay

In the transwell cell migration assay, 24-well transwell plates with 8 μm pore size inserts (MCSP24H48, Millipore, Burlington, VT, USA) were used. HCAEC cells were re-suspended in an Endothelial Cell Growth Medium (ECM, Lonza Walkersville, Walkersville, MD, USA) without serum. A total of 2 × 10^5^ cells were seeded onto the upper chamber (insert), while ECM supplemented with 5% FBS was added to the lower chamber, followed by incubation at 37 °C in 5% CO_2_ for 16 h. Then, a cotton tip was applied to remove the media and remaining cells that did not migrate down to the bottom side of the insert membrane. After that, the membranes were fixed with 100% methanol for 30 min, washed with PBS, and stained with crystal violet (Sigma, Burlington, VT, USA) for 30 min. For each insert, cells in the cross line forming 4 fields under a 100-fold magnification were counted. In addition to direct counting cells, we also repeated the assays with the stained cells that were collected and dissolved in 10% acetic acid and measured at a wavelength of 590 nm by the ELISA reader.

### 4.7. Permeability Assay

The trans-well (MCSP24H48, Millipore, Burlington, VT, USA) kit, composed of the upper chamber and the lower chamber, was applied to examine the permeability of the endothelial layer. First of all, 2 × 10^5^ endothelial cells (HCAECs) were seeded in the upper chamber for 24 h, with the upper chamber and the lower chamber containing a 200 μL and 600 μL culture medium, respectively. Then, the endothelial cells were treated with S100A4 recombinant proteins (4137-S4-050, R&D Systems, Minneapolis, MN, USA) or DMSO. After 24 h of treatment, the HCAEC-seeded upper chamber was washed with PBS three times. Then, the trypan blue infiltration (referring to a previous study [43]) and protein infiltration assays were applied to examine the permeability of HCAECs. In the trypan blue infiltration assay, the upper chamber was added with 100 μL of trypan blue dye (03-102-1B, Biological industries, Kibbutz Beit Haemek, Israel), and we observed how much time was needed for the trypan blue to penetrate the endothelial layer and move to the lower chamber. In a protein infiltration assay, the upper chamber was added with 100 μL or 20% FBS. After 30, 60, 90, and 120 min, we collected a 10 μL solution from the lower chamber, followed by measuring the protein concentration.

## 5. Conclusions

Our study demonstrates that both up-regulated S100A4 and down-regulated CAT increased the risk of CAL in KD. Further in vitro studies concluded that S100A4 treatment resulted in an incompact endothelial layer and made HCAECs more susceptible to in vitro neutrophil infiltration. Therefore, S100A4 could be a potential therapeutic target for CAL in KD.

## Figures and Tables

**Table 1:** Demographic data of subjects.

Factors/Category	CAL+KD Patients (n = 32)	CAL-KD Patients (n = 31)	*p*-Value
Age (years)	1.9±1.4	1.7 ± 1.0	0.61
Sex (male)	18	16	0.71
Inpatient day	9.5 ± 4.4	7.2 ± 3.9	0.03
Height (cm)	83.6 ± 15.9	82.3 ± 13.2	0.74
Weight (kg)	11.8 ± 3.7	10.9 ± 2.6	0.26
Z-LCA	2.5 ± 0.7	0.9 ± 0.6	6.04 × 10^−15^
Z-RCA	2.1 ± 1.6	1.4 ± 0.7	4.29 × 10^−3^

CAL—coronary artery lesion; KD—Kawasaki disease; Z-LCA—Z score of left coronary artery; Z-RCA—Z score of right coronary artery.

## Data Availability

Not applicable.

## Supplementary Materials

The supporting information can be downloaded at: https://www.mdpi.com/article/10.3390/ijms232112770/s1.
